# Supplementary material for: A Nomogram for Predicting the Possibility of Peripheral Neuropathy in Patients with Type 2 Diabetes Mellitus
Source: Brain Sci. 2022 Sep 30;12(10):1328. doi: 10.3390/brainsci12101328 (PMC9599450; doi:10.3390/brainsci12101328)
Supplement: Supplementary file 1 [file brainsci-12-01328-s001.zip › brainsci-1901639-supplementary.pdf]

Table S1. Correlation analysis of variables with nerve conduction studies parameters in total patients.

	F-wave		MNAmp		MNCV		SNAmp		SNCV	
	r	*P*	r	*P*	r	*P*	r	*P*	r	*P*
Age (year)	−0.05	0.162	−0.34	<0.001	−0.07	0.043	−0.25	<0.001	−0.11	0.003
Duration (year)	0.01	0.883	−0.25	<0.001	−0.13	<0.001	−0.27	<0.001	−0.17	<0.001
BMI (kg/m^2)	−0.07	0.050	−0.01	0.806	0.05	0.171	−0.02	0.657	0.07	0.047
SUA (umol/L)	0.03	0.441	−0.03	0.495	−0.07	0.040	−0.02	0.588	−0.05	0.180
HbA1c (%)	0.09	0.015	−0.05	0.227	−0.08	0.027	0.01	0.755	−0.06	0.116
FT3 (pmol/L)	0.05	0.205	0.23	<0.001	0.16	<0.001	0.10	0.019	0.10	0.006

MNAmp, mean motor nerve amplitude; MNCV, mean motor nerve conduction velocity; SNAmp, mean sensory nerve amplitude; SNCV, mean sensory nerve conduction velocity.

Table S2. Multivariate linear regression analysis of the relationship between variables and nerve conduction parameters in total patient

	MNAmp		MNCV		SNAmp		SNCV	
	β	P	β	P	β	P	β	P
Sex	−0.281	0.263	−2.431	< 0.001	−6.563	< 0.001	−3.411	< 0.001
Age (year)	−0.080	< 0.001	−0.040	0.137	−0.270	< 0.001	−0.081	0.055
Duration (year)	−0.082	< 0.001	−0.162	< 0.001	−0.423	< 0.001	−0.327	< 0.001
BMI (kg/m^2)	−0.009	0.794	0.077	0.379	−0.173	0.277	0.189	0.044
SUA (umol/L)	−0.002	0.179	−0.006	0.047	−0.002	0.737	−0.006	0.197
HbA1c (%)	−0.213	< 0.001	−0.420	< 0.001	−0.533	0.016	−0.546	0.004
FT3 (pmol/L)	0.680	< 0.001	1.443	< 0.001	0.971	0.234	0.919	0.122

MNAmp, mean motor nerve amplitude; MNCV, mean motor nerve conduction velocity; SNAmp, mean sensory nerve amplitude; SNCV, mean sensory nerve conduction velocity.
